# GA-OH enhances the cytotoxicity of photon and proton radiation in HPV^+^ HNSCC cells

**DOI:** 10.3389/fonc.2023.1070485

**Published:** 2023-02-10

**Authors:** Lennox Chitsike, Antonella Bertucci, Marcelo Vazquez, Steve Lee, Juli J. Unternaehrer, Penelope J. Duerksen-Hughes

**Affiliations:** ^1^ Department of Basic Sciences, Loma Linda University School of Medicine, Loma Linda, CA, United States; ^2^ Department of Radiation Medicine, Loma Linda University School of Medicine, Loma Linda, CA, United States; ^3^ Department of Otolaryngology & Head/Neck Surgery, Loma Linda University School of Medicine, Loma Linda, CA, United States; ^4^ Division of Biochemistry, Department of Basic Sciences, Loma Linda University, Loma Linda, CA, United States

**Keywords:** radiotherapy, protons, photons, radio-sensitization, HPV+OPSCC, HPV- OPSCC, E6, de-escalation

## Abstract

**Introduction:**

Treatment-related toxicity following either chemo- or radiotherapy can create significant clinical challenges for HNSCC cancer patients, particularly those with HPV-associated oropharyngeal squamous cell carcinoma. Identifying and characterizing targeted therapy agents that enhance the efficacy of radiation is a reasonable approach for developing de-escalated radiation regimens that result in less radiation-induced sequelae. We evaluated the ability of our recently discovered, novel HPV E6 inhibitor (GA-OH) to radio-sensitize HPV+ and HPV- HNSCC cell lines to photon and proton radiation.

**Methods:**

Radiosensitivity to either photon or proton beams was assessed using various assays such as colony formation assay, DNA damage markers, cell cycle and apoptosis, western blotting, and primary cells. Calculations for radiosensitivity indices and relative biological effectiveness (RBE) were based on the linear quadratic model.

**Results:**

Our results showed that radiation derived from both X-ray photons and protons is effective in inhibiting colony formation in HNSCC cells, and that GA-OH potentiated radiosensitivity of the cells. This effect was stronger in HPV+ cells as compared to their HPV- counterparts. We also found that GA-OH was more effective than cetuximab but less effective than cisplatin (CDDP) in enhancing radiosensitivity of HSNCC cells. Further tests indicated that the effects of GA-OH on the response to radiation may be mediated through cell cycle arrest, particularly in HPV+ cell lines. Importantly, the results also showed that GA-OH increases the apoptotic induction of radiation as measured by several apoptotic markers, even though radiation alone had little effect on apoptosis.

**Conclusion:**

The enhanced combinatorial cytotoxicity found in this study indicates the strong potential of E6 inhibition as a strategy to sensitize cells to radiation. Future research is warranted to further characterize the interaction of GA-OH derivatives and other E6-specific inhibitors with radiation, as well as its potential to improve the safety and effectiveness of radiation treatment for patients with oropharyngeal cancer.

## Introduction

Head and neck squamous cell carcinomas (HNSCCs) are responsible for about 650,000 new cases and 300,000 deaths every year worldwide (1). These cancers arise from anatomical regions of head and neck that include the oral cavity, oropharynx, hypopharynx, larynx, and nasopharynx (1). They can be divided into two distinct categories, oropharyngeal squamous cell carcinoma (OPSCC) that is related to HPV, and HPV-unrelated cancers that are driven by carcinogens such as tobacco and alcohol. Even though HPV-related OPSCC responds better to treatment and carries better prognosis compared to its HPV^-^ counterpart, differential oncologic treatment of HPV^+^ is evolving. (2–6). Surgical resection or external beam radiation with chemo- or targeted-therapy is the standard of care for locally advanced HNSCC.

Ionizing radiation primarily targets the cellular DNA and causes a number of lesions that include base damages, single-strand breaks (SSBs) and double-strand breaks (DSBs). DSBs are the most lethal if not repaired (7, 8). Proteins such as ATM initiate the DNA damage response (DDR) through posttranslational modifications of histone H2A and subsequent recruitment of a host of factors (53BP1, CHK1, CHK2, p53, p21 etc.) that activate cell cycle checkpoint and repair. DDR proteins are responsible for coordinating the repair of DSBs through a number of repair pathways (8–10). If the damage is not repaired, radiation-induced cell death may occur through apoptosis, necrosis, mitotic catastrophe and senescence (8, 9). In general, HPV^+^ cells have been observed to exhibit higher radiosensitivity compared to HPV^-^ cells due to differentials in DNA repair capacity, p53 and cell cycle regulation among other factors (11, 12). Because of this enhanced radiosensitivity, one of the most significant ongoing challenges with radiation treatment in HNSCC is not necessarily efficacy but rather long term-toxicity. The intensified standard radiation regimen that is deemed necessary for therapy often results in significant sequelae that negatively affect basic human functions such as talking, eating, breathing, hearing etc. (13, 14). However, because HPV-associated OPSCC has excellent outcomes, many now believe that the current standard dose is resulting in overtreatment of the HPV^+^ patient cohort. Numerous endeavors to de-escalate the radiation treatment regimens are already underway with a goal of lowering toxicity without compromising clinical outcomes (3, 11, 15–18). Most de-escalation attempts thus far have revolved around limiting the toxicity inflicted by photon-based radiotherapy.

Proton therapy is another option currently being considered for de-escalation, and the specific characteristic of this type of radiation prime it for success with a de-escalated approach. Proton radiation has low entrance and exit doses, and deposits maximum energy at the Bragg peak followed by a sharp drop in energy deposition at the distal fall-off. These unique characteristics spare proximal normal tissue and organs at risk (OAR) (19, 20). Moreover, protons have higher linear energy transfer (LET) around the Bragg peak and produce ionization events that are more clustered per given dose than are photons. Clustered ionizations produce DSBs that are more complex and difficult to repair and generally result in higher cytotoxicity per unit dose, which may explain the observed higher relative biological effectiveness (RBE) over photons (19–21). Additionally, another strategy employing radiosensitization still under development is the use of targeted therapy in combination with radiation (22, 23). Thus far, cisplatin and cetuximab are used clinically as conventional radiosensitizers in chemoradiotherapy (CRT) regimens, but neither is ideal for a number of reasons. Chief among them is that cisplatin exacerbates the toxicity of radiation (23) and cetuximab, which has a safer profile that cisplatin, has been found to have inferior survival outcomes as a radiosensitizer in most recent clinical trials as compared to cisplatin (15–17, 24). These observations call for the discovery of novel agents that are both safer and more effective in synergizing with radiation for successful de-escalated chemoradiotherapy regimens.

We previously discovered an E6 inhibitor, GA-OH, and demonstrated that it can rescue pro-apoptotic molecules, including p53, and promote apoptosis (25). We therefore tested the efficacy of GA-OH in combination with radiation to evaluate radio-potentiation. Specifically, we studied the effects of GA-OH together with radiation on clonogenic survival, DSB repair, apoptosis and cell cycle distribution in both HPV^+^ and HPV^-^ HNSCC cell lines and compared the radiosensitizing effects of GA-OH, cisplatin and cetuximab. We further investigated whether some of the effects we observed in cell lines can be re-capitulated in clinically more relevant primary tumor cells.

## Materials and methods

### Cell culture

HNSCC cell lines were obtained from several sources: UM-SCC47-TC-Clone 3 (#47CL3), UM-SCC 19, UM-SCC 29, and UMSCC 104 were a gift from Dr. Thomas Carey, University of Michigan (Michigan, USA). The SCC 84 cell line was a gift from Dr. John Lee, Sanford Research (South Dakota, USA). UPC1-SCC152 was purchased from ATCC. The HNSCC cells were cultured in Dulbecco’s Modified Eagle Medium (DMEM, Mediatech, Manassas, VA, USA) supplemented with 10% of FBS.

### Establishing primary tumor cell lines

Patient samples (n=4) were obtained from the Loma Linda University School of Medicine, Department of Otolaryngology and Head/Neck Surgery through the Loma Linda University Tissue Biorepository. Patients were all males with ages ranging from 65 to 77 years and the tissues were obtained following laryngectomy and glossectomy. The freshly resected tissues were kept on ice until ready for processing. Samples were transferred to petri dishes and washed with PBS-2X Gentamicin 3 times. Samples were minced using a sterile razor blade until they appeared as puree. The puree was passed over a 70 µm strainer using a plunger from a 3 mL syringe. Plain DMEM was used to wash the cells. The cells were then centrifuged at 1500 rpm for 10 mins. If pellet was red, red blood cells were removed using Ficoll. Otherwise, cells were counted using an automated cell counter and resuspended in three parts Ham’s F12, one part DMEM (Fisher Scientific) supplemented with 5% FBS (Omega Scientific), 10uM insulin, 0.4uM hydrocortisone, 2ug/ml isoprenaline, 24ug/ml adenine (chemicals from Sigma-Aldrich), 100U/ml penicillin, 10ug/ml streptomycin (Fisher Scientific). 5-10 uM Y27632 (BioGems) was added to establish growth *in vitro* (26). Cells were plated at 2 X 10^6^ in 6 well plates. Cells were monitored over time for development of clones that were then used to establish primary cultures.

### Radiation devices and dose measurements

Cell irradiations with protons and photons (x-rays) was completed at the James M. Slater Proton Treatment and Research Center, Loma Linda California. Proton radiation exposures were done at room temperature. 250 MeV protons were modulated to generate a 5.0 cm wide spread-out Bragg peak (SOBP). The cells were located at a water equivalent depth of 29.6 cm, specified using CIRS plastic water blocks, which placed the cells in the uniform dose SOBP region of the proton dose profile. Irradiations were conducted with the beam incident on the underside of the flask to ensure accurate placement of the cell layer with respect to the proton depth dose profile. The proton field size employed for the irradiation of the HNSCC cell lines was circular with an 18 cm diameter. Protons were delivered from our synchrotron accelerator in a pulsed fashion, with a pulse duration of 0.125 seconds and a duty cycle of 2.2 seconds. This pulsed modality of beam delivery gave a dose rate of approximately 0.8 Gy/min, and cells were exposed to single doses of 0, 0.5, 1, 2, 4, 8 Gy. For photon (x-rays) irradiations, a 22 MeV TrueBeam linear accelerator (Varian Medical System, Palo Alto, CA) was employed to expose cells with single doses of 0, 1, 2 and 4 Gy with a dose rate of 3 Gy/min at room temperature. The x-ray field size employed for the irradiation of the HNSCC cell lines was square beam spot of 20 x 20 cm.

### Western blot analysis

Attached cells treated with inhibitor and/or radiation were washed with 1X ice cold PBS 24 hours post-treatment. Protein lysate buffer containing a cocktail of protease inhibitors and cells were scraped off into a tube on ice. The cells were incubated on ice for 10 minutes. Cell lysates were separated by SDS-PAGE and electrophoretically transferred to PVDF membranes. The membranes were blocked before anti-caspase 8 (Cell signaling), p53 (Cell Signaling), cleaved PARP (Cell Signaling), cleaved caspase 3 (Cell signaling), p21 (Cell Signaling), 53BP1 (Cell Signaling). and β-actin (Cell signaling) were applied at 1:5000 dilution. Anti-mouse and anti-rabbit secondary antibodies were then employed (LI-COR Biosciences, Lincoln, NE, USA). Signals were measured using the Odyssey Infrared Imaging system (LI-COR Biosciences) and quantified using LICOR software.

### MTT cell viability assays

All working concentrations of GA-OH were diluted to the desired concentration in PBS. To test the effect of GA-OH on viability, all primary cells were seeded at 2 × 10^4^ per well in 96-well plates and allowed to attach overnight. GA-OH was added to the cells following serial dilution and the cells incubated at 37 °C for 24 hr. Viability was then measured using the MTT assay, performed as described previously (27). The experiments were repeated at least three times on 3 different days. Data presented are from a representative experiment. Cell viability and potency were assessed from % inhibition relative to the vehicle control, and IC_50_ dose curves were generated using GraphPad Prism.

### Clonogenic survival

Sub-confluent monolayer cells were trypsinized and counted using an automated cell counter. Cells were re-suspended and plated into 6 well plates in DMEM at cell densities ranging from 250-1,000 depending on the cell line. Cells were exposed to X-ray or proton radiation beams (0, 1, 2, 3 or 4 Gy) with or without addition of GA-OH, cisplatin or cetuximab 1-hour prior to radiation treatment. After irradiation, the medium was changed and cells were then allowed to grow for 10-20 days, depending on the cell line, before fixing and staining. A mixture of methanol/acetic acid was used for fixing, followed by 0.5% crystal violet and the number of colonies with more than 50 cells were counted. Surviving fractions were determined by dividing the number of colonies by the number of cells seeded as a product of the corresponding plating efficiency (PE). PE values for SCC19, SCC29, SCC84, SCC47, SCC104 and SCC152 were 0.30, 0.25, 0.29, 0.15, 0.19 and 0.08, respectively. GraphPad Prism was used for plotting survival fractions curves and analyzing them using the linear quadratic model. For the isoeffective doses of GA-OH, cisplatin and cetuximab in this study, curves of survival fractions of cells exposed to varying concentrations of the three inhibitors were first plotted using non-linear regression and then IC_20_ values were calculated from the Hillsope using Graphpad Prism.

### DNA damage and repair studies

Sub-confluent monolayer cells were trypsinized and counted using an automated cell counter. Cells were re-suspended and plated into 1-well cell culture glass chamber slides (Nest Scientific, USA) in DMEM at cell densities of 10, 000 cells/well. The glass chambers were put on trays and incubated overnight. Cells were exposed to X-ray radiation with or without addition of GA-OH 1 hour prior and incubated for either 30 mins or 24 hours before fixing them with ice cold methanol. Cells were then permeabilized with 0.5% Triton-X 100 and blocked with 5% goat serum anti-53BP1 (1:100, Cell signaling) primary antibodies were added for 1 hr. After washing, appropriate secondary antibody (Alexa 488-invitrogen) was added and slides were later washed and counterstained with DAPI (Vectashield). Immunoreactions were visualized and imaged using Leica microscopy and the number of foci was independently counted manually four times. The average counts were then calculated and used to report the number of foci.

### Annexin V analysis

Treated and untreated cells were trypsinized after 24 hours and resuspended in media before being counted with a cell counter. About 1.5 X 10^5^ cells were added to round bottomed 96 well plate and centrifuged for 5 minutes at 1500 rpm at 4°C. Supernatant was removed and cells were washed with PBS. 10 µL of 1X Annexin V binding buffer was added plus 0.5 µL of Annexin V stain. The plates were incubated for 15 minutes at 4°C in the dark. 1 µL of 7AAD was then added and plates were incubated for an additional 5 mins. Unstained, Annexin V only and 7AAD only control were also included. 180 µL of Annexin V buffer was added to all wells and centrifuged for 5 min at 1500 rpm. Supernatant was removed and cells were resuspended in 100 µL of 1% PFA. Cells were kept in the dark at 4°C until they were ready to be analyzed using a MaxQuant flow cytometer. FCS files of the data were analyzed and processed using FlowJo 10.7.1 software. Establishment of gates and quadrants was guided by the unstained control.

### Cell cycle analysis

Cells for cell cycle analysis were initially prepared as for flow cytometry. Once counted, about 3 million cells were resuspended in microcentrifuge tubes and centrifuged for 5 min at 2000 rpm. The cells were then washed in PBS and fixed with ice cold 70% ethanol dropwise. The cells were then transferred to -20°C for >1hr. Thereafter the cells were warmed to room temperature and centrifuged at 2000 rpm for 5 mins and washed with PBS. 0.5 mL of Propidium iodide/RNAse A staining buffer (Invitrogen) was added and samples were incubated at room temperature for 30 mins in the dark. Cells were analyzed using MasQuant flow cytometer analyzer within 3 hours. FCS files of the data were analyzed and processed using FlowJo 10.7.1 software. Pulse processing was done on FlowJo to exclude doublets from the analysis, and singlets populations were then used to obtain the proportions of cells in the respective cell cycle phases.

### qPCR

Cells were treated with radiation with or without GA-OH and harvested after 24 hours. Total RNA was extracted from cells using the Trizol reagent (RNA Zymo) and the Invitrogen RNA extraction protocol. cDNA was synthesized using a BioRad iScript (BioRad), as per manufacturer’s instructions. qRT-PCR was performed using iScript SYBR Green Mix (BioRad) on the BioRad PCR machine. Sequences of primers for amplifying p53, p21, Bax, PUMA, NOXA and GAPDH mRNA were obtained from previously described primers (28–30) and obtained from IDT. The oligonucleotides sequences for the gene primers were as follows: p53: Forward 5’- CTG CTC AGA TAG CGA TGG TCT C- 3’ and Reverse 5’- TTG TAG TGG ATG GTG GTA CAG TCA-3’; p21: Forward 5’-GGC AGA CCA GCA TGA CAG ATT-3’ and Reverse 5’- GCG GAT TAG GGC TTC CTC TT-3’; PUMA: Forward 5’- GAC GAC CTC AAC GCA CAG TA-3’ and Reverse 5’- GGA GTC CCA TGA TGA GAT TGT-3’; NOXA: Forward 5’- CAG GAC TGT TCG TGT TCA GC-3’and Reverse 5’- TTC TGC CGG AAG TTC AGT TT-3’; Bax: Forward 5’- AGC GGC GGT GAT GGA C-3’ and Reverse 3’- AAA AGG GCC CCT GTC TTC AT-3’; GAPDH: Forward 5’- GCA CCG TCA AGG CTG AGA AC-3’ and Reverse 5’- ATG GTG GTG AAG ACG CCA GT-3’. Relative target mRNA levels were determined using the 2^−(ΔCt)^ method (31), and were expressed as the ratio to GAPDH mRNA.

### Formulas and calculations

Survival Fraction (SF): [(# of colonies/number of plated cells) with irradiated]/[(# of colonies/# of plated cells without radiation)]

Relative Biological Effectiveness (RBE) at 0.1 SF: Dose of photons (X-ray) given/Dose of protons given (for same cell survival rate)

Dose enhancement ratio (DER)=Dose of photons (X-rays)/Dose of photons (X-rays) plus GA-OH

### Statistics

All experimental data presented here are means ± standard deviation of the mean. Experiments were conducted in replicates and then repeated at least thrice unless stated otherwise. Student’s t-tests were employed to compare the means of two groups of independent samples for different parameters such as cell death-induction and numbers of 53BP1 foci. *p*< 0.05 was used as the threshold for statistical significance.

## Results

### Radiosensitivity of HNSCC cell lines to standard treatments

**Figure 1:**
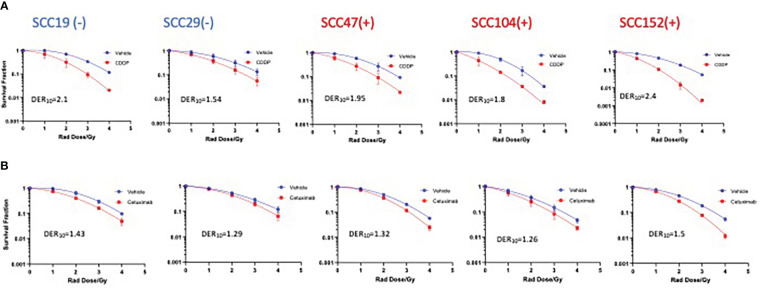
Sensitization of HNSCC cells to X-ray radiation combined with CDDP and Cetuximab. HPV^-^ **(-)** and HPV^+^(+) cells were treated with either CDDP or cetuximab (see **Table S1** for concentrations) in combination with radiation (0-4Gy)). Colonies were counted after 14-21 days of incubation and normalized to controls. Survival analysis was performed using the linear quadratic model and GraphPad. Both CDDP **(A)** and cetuximab **(B)** show sensitization in all cell lines regardless of HPV status. Values shown represent means ± standard deviation from 3 independent experiments.

To gain insights on the baseline levels of our cell line responses to radiation, we began the study by first assessing how efficient clinically approved drugs that are used in combination with radiation are in improving the effectiveness of photon radiation. Cisplatin is the standard regimen for chemoradiotherapy, while cetuximab is the alternative in patients with certain pre-existing co-morbidities. To this end we examined the clonogenic potential of 2 HPV^-^ (SCC19 and SCC29) and 3 HPV^+^ (SCC47, SCC194, SCC152) cell lines treated with cisplatin or cetuximab together with photon radiation. The concentrations of the agents used in this study are shown in **Table S1**. The selected doses in **Table S1** represent the dose needed to reduce colony formation by about 20% and using these similar potencies would enable radio-sensitization head to head comparisons. When radiation was used alone, HPV^+^ cells were relatively more radiosensitive as compared to HPV^-^ cells (**Figure 1**). Specifically, HPV^+^ cells exhibit lower survival fractions at every dose of radiation given. This is further illustrated by survival fraction at 2Gy (SF2), a surrogate of intrinsic radiosensitivity, which is again lower for HPV^+^ cells (**Supplementary Figure 1**). Addition of cisplatin significantly improved the effectiveness of radiation in all cell lines, irrespective of HPV status (**Figure 1A**). This can be highlighted by looking at the extent of sensitization when cisplatin is added. The highest DER_10_ values were 2.1 and 2.4 for SCC19 and SCC152 (**Table 1**), respectively. We performed similar experiments with cetuximab and found that cetuximab also demonstrated the ability to improve the effectiveness of radiation. Like cisplatin, cetuximab exhibited no clear discrimination between HPV^-^ and HPV^+^ cells (**Figure 1B**). However, the effect of cetuximab was reduced compared to cisplatin, with the highest DER_10_ value of only 1.43 for SCC19 (**Table 1**).

**Table 1:** Dose enhancement ratios (DER_10_) of X-ray photon radiation with either CDDP, GAOH or cetuximab calculated at SF of 0.1.

CDDP + Photon Radiation		GA-OH + Photon Radiation		Cetuximab + Photon Radiation	
Cell Line	DER_10_	Cell Line	DER_10_	Cell Line	DER_10_
SCC19(-)	2.1	SCC19(-)	1.19	SCC19(-)	1.43
SCC29(-)	1.54	SCC29(-)	1.21	SCC29(-)	1.29
SCC47(+)	1.95	SCC47(+)	1.7	SCC47(+)	1.32
SCC104(+)	1.8	SCC104(+)	1.59	SCC104(+)	1.26
SCC152(+)	2.4	SCC152(+)	1.66	SCC152(+)	1.5

### Effect of GA-OH on radio-cytotoxicity as compared to cisplatin and cetuximab

**Figure 2:**
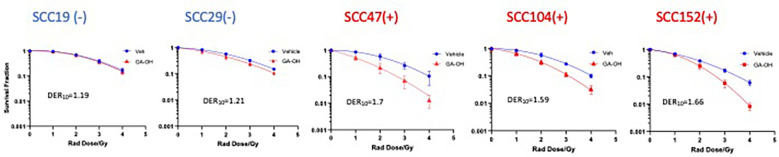
Sensitization of HNSCC cells to X-ray radiation combined GA-OH. HPV^-^ (-) and HPV^+^(+) cells were treated with GA-OH (see **Table S1** for concentrations) in combination with radiation (0-4Gy)). Colonies were counted after 14-21 days of incubation and normalized to controls. Survival analysis was performed using the linear quadratic model and GraphPad. GA-OH sensitization of radio cytotoxicity is biased more towards HPV^+^ cells. Values shown represent means ± standard deviation from 3 independent experiments.

**Figure 3:**
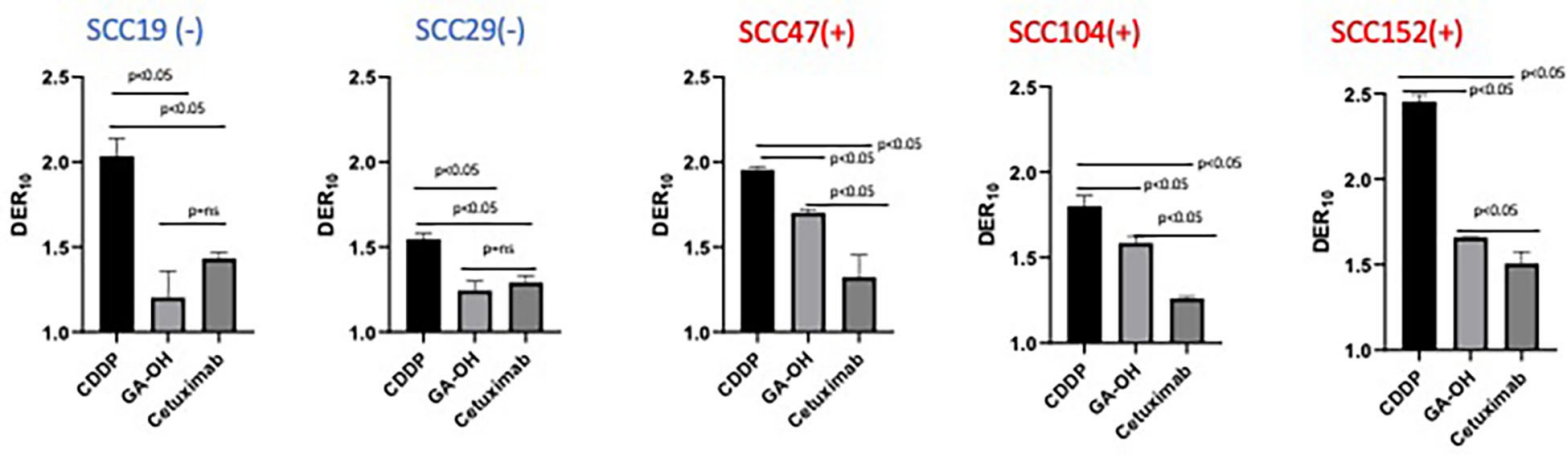
Relative degree of X-ray radio-sensitization by CDDP, GA-OH and cetuximab. Dose enhancement ratios (DER_10_) of the 3 agents were determined at a cell surviving fraction (SF) of 0.1. Generally, CDDP shows the greatest sensitization and cetuximab shows the least sensitization, with no discrimination between HPV^+^ and HPV^-^ cell lines. GA-OH shows greater sensitization in HPV^+^ cell lines than does cetuximab, though the magnitude of sensitization is less than that of CDDP. Values shown represent means ± standard deviation from 3 independent experiments.

We previously identified an E6 specific inhibitor, GA-OH, from a high content screen and have shown that it increases caspase 8 and p53 levels in HPV^+^ cells (25). Given that expression of wildtype p53 has been identified as a critical biomarker for radiosensitivity in HNSCC and the role caspase 8 plays in apoptosis, we first investigated how GA-OH modulates radiosensitivity of 2 HPV^-^ and 3 HPV^+^ cell lines. We assessed cellular response to X-ray radiation using the colony formation assay. The addition of GA-OH overall did not significantly affect the effectiveness of photon irradiation in the two HPV^-^ cell lines, SCC19 and SCC29 (**Figure 2**). This is evidenced by slight enhancement of the dose ratios calculated; the DER_10_ values were 1.19 and 1.21 for SS19 and SCC29 (**Table 1**), respectively. On the other hand, GA-OH notably sensitized the response of all 3 HPV^+^ cell lines to radiation (**Figure 2**). The DER_10_ values of SCC47, SCC104 and SCC152 were calculated to be 1.79, 1.59 and 1.62, respectively (**Table 1**). These results overall showed the first evidence of HPV-dependency of GA-OH towards radiosensitivity. In comparison, GA-OH was less effective as a radiosensitizer than cisplatin as measured by survival fractions and DER_10_ values (**Figure 3**, **Table 1**). However, GA-OH was more effective than cetuximab. The effects of these 3 agents and their relative efficacies are quantitatively summarized in **Figure 3** and **Table 1**.

### Effect of GA-OH on the sensitivity of HNSCC cells to proton radiation

**Figure 4:**
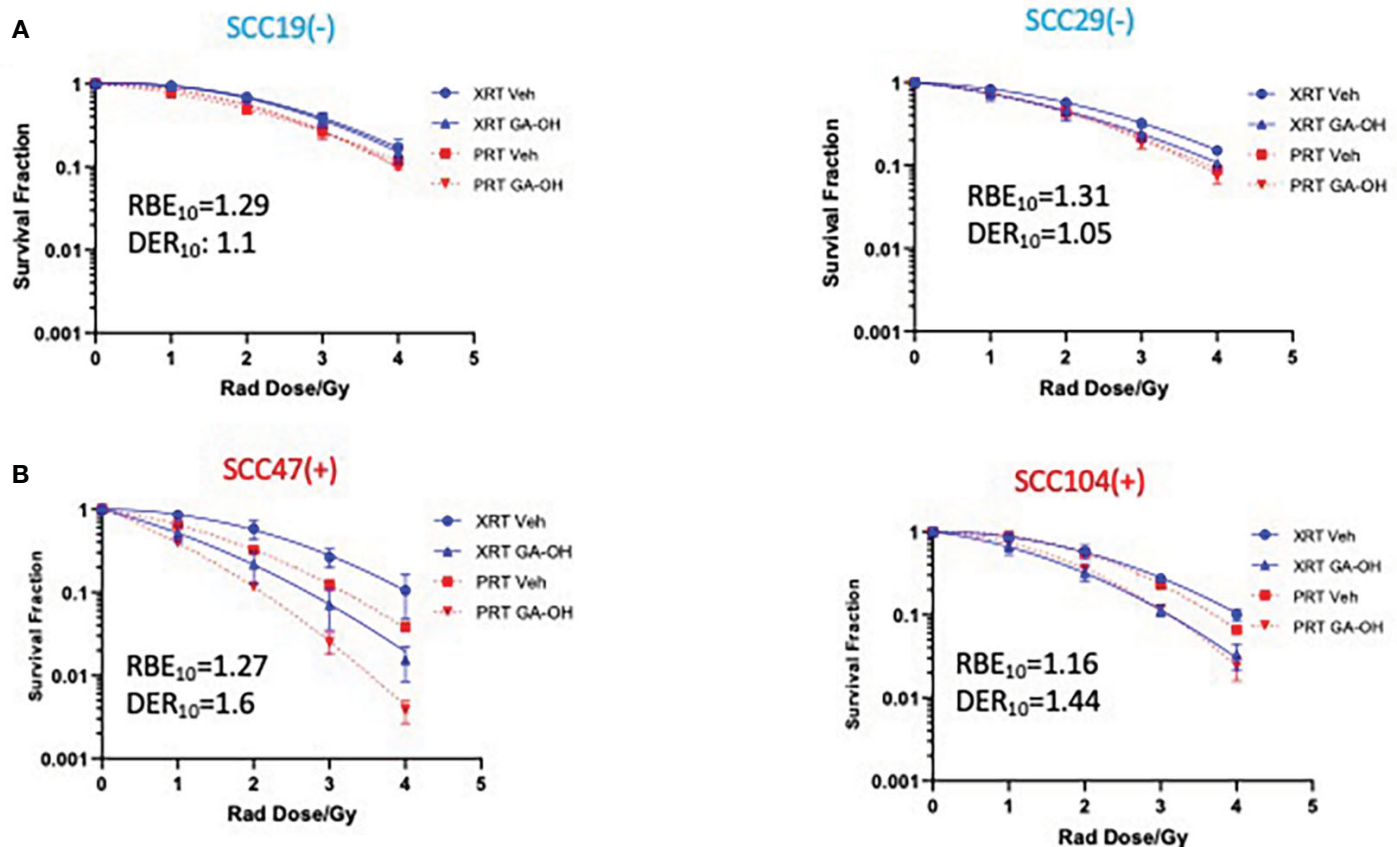
Sensitization of HNSCC cells to X-ray versus proton radiation combined with GA-OH. HPV^-^ **(-)** and HPV^+^(+) cells were treated with GA-OH (see table S1 for concentrations) in combination with X-ray radiation or protons (0-4Gy)). Colonies were counted after 14-21 days of incubation and normalized to controls and survival analysis was performed using the linear quadratic model and GraphPad. Relative effectiveness of protons compared to X-ray (photons) with and without GA-OH by way of RBE_10_ calculations in HPV^-^ **(A)** and HPV^+^ **(B)** cells. Values shown represent means ± standard deviation from 3 independent experiments.

We next investigated how GA-OH might regulate the response of HNSCC cells to proton, as well as to photon, radiation. Proton therapy is currently being evaluated as a de-escalation treatment alone (15–17, 24), and agents that enhance the efficacy and selectivity of protons will serve to increase the prospects of proton therapy as an approved de-intensified modality in the future. To explore this potential, we subjected 2 HPV^-^ and 2 HPV^+^ cell lines to the colony formation assay. Overall, protons were generally more effective in suppressing the viability of cells compared to photons as evidenced by lower survival fractions per any given dose (**Figure 4**). These findings are reflected in the relative biological effectiveness (RBE) values (calculated without GA-OH), as each value is higher than 1 for all cell lines tested (**Table S2**). Currently, proton beam therapy in clinical use is prescribed based on a constant relative biological effectiveness (RBE) of 1.1 relative to X-ray photons (Wang, 32). Our results show RBEs above this constant, with values ranging from 1.16 to 1.31. A closer examination of the two experiments with protons and photons showed similar overall trends of the radio-response between HPV^+^ and HPV^-^ cell lines. First, HPV^-^ cells were intrinsically relatively less sensitive to protons than were HPV^+^ cells, as was observed with photons (**Figure 4**, **Supplementary Figure 1**). Second, HPV^-^ cell lines also did not show an appreciable increase in radiosensitivity when GA-OH was added (**Figure 4**). Third, HPV^+^ cell lines (SCC47 and SCC104) were significantly sensitized to protons following treatment with GA-OH as reflected by their DER values (**Figure 4**). However, the relative magnitude of radio-sensitization of HPV^+^ cells to GA-OH was smaller with protons than that we observed with photons. Specifically, we found that GA-OH did not improve the RBE values of protons for either HPV^-^ or HPV^+^ cell lines (**Table S2**) compared to the vehicle. In other words, GA-OH does improve the radio-toxicity of protons to cells as compared to protons alone, but its relative effects of sensitization are larger with X-ray photons than with protons (**Table S2**).

### Resolution of radiation-induced DSBs following GA-OH treatment

**Figure 5:**
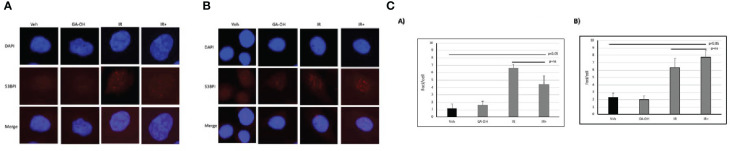
Effects of GA-OH on radiation-induced nuclear 53BP1 foci post-irradiation. HPV^-^ **(A)** and HPV^+^ **(B)** cells were stained with anti-53BP1 and counter-stained with DAPI following treatment with GA-OH and X-ray radiation. 53-BP1 foci represent residual DSBs and representative images of nuclei of HPV^-^ cells and HPV^+^ cells after treatment are shown in **(A, B)**. **(C)** shows quantification of 53BP1 foci. Blue represents DAPI counter-stain and red 53BP1 foci.

To begin to understand the mechanisms contributing to the observed effects of GA-OH on the cellular radio-response, we investigated how cells respond to DSB DNA damage. We first analyzed DSB repair through the assessment of foci of 53BP1, a known marker for unresolved DSBs, at 24 hours post-irradiation (IR). We used a 4 Gy dose of irradiation in two of the same cell lines as described above (SCC19 and SCC47) and determined residual DNA damage after the initial exposure (**Figure 5**). There were some background foci in the nuclei of untreated cells for both SCC19 and SCC47, with SCC47 displaying slightly more. The effect of GA-OH on the foci formation was minimal and about the same as the background. This may be because GA-OH is not known or expected to have direct DNA damaging effects. The application of radiation to the cells, on the other hand, showed more significant induction of foci. For SCC19, the number of residual foci increased about 6-fold with radiation alone. Interestingly, the addition of GA-OH was associated with a decrease in the number of observed foci (**Figure 5**), even though the decrease was not significant. We had hypothesized close to similar levels of residual DSBs since colony formation assay had shown only a slight improvement in the decrease of survival with GA-OH. It is not clear whether these observations with the DNA damage assay are due to the protective effects of GAOH at low concentrations in HPV^-^ cells, or to some unique DNA repair mechanism in SCC19. For the SCC47 cell line, treatment with radiation also led to significant foci remaining 24 hours post-irradiation as compared to either the vehicle or GA-OH alone. With the addition of GA-OH to radiation, we observed an increase of persistence of foci post-irradiation compared to radiation alone, although this increase was not statistically significant (**Figure 5**). This result in SCC47 cells may suggest that GA-OH can, albeit weakly, interfere with the ability of cells to resolve the breaks.

### GA-OH effect on radiation-induced G2/M arrest

**Figure 6:**
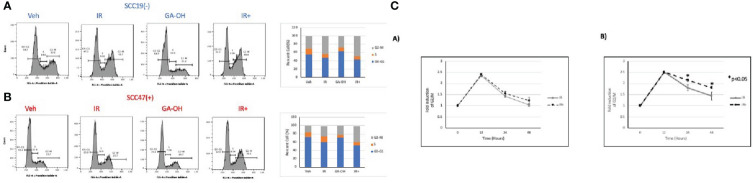
Cell cycle changes after radiation and GA-OH treatment. SCC19 **(A)** and SCC47 **(B)** cells treated with or without GA-OH were stained with propidium iodide 24 hours post-irradiation and analyzed using flow cytometry. In both cell lines, G2-M is the most affected stage of cell cycle and there is an increase in G2-M arrested cells. SCC47 has a higher proportion of cells that are still arrested in G2-M phase after 24 hrs compared to SCC19. **(C)** Relative temporal changes of the G2-M fraction of cells over a time period of 48 hours in SCC19 (left) and SCC47 (right). Values shown represent means ± standard deviation from 3 independent experiments.

We also looked at the effect of GA-OH on the distribution of cell cycle phases following irradiation by analyzing the DNA content in the different stages of the cell cycle using propidium iodide staining. An initial look at the distribution of cells in cell cycle at 24 hours showed that G2/M was the phase with the most significant change upon irradiation (**Figure 6**). GA-OH by itself caused an appreciable increase of cells the G1 phase with a concomitant reduction of S phase cells. In both HPV^-^ and HPV^+^ cells, there was a significant increase in G2/M-arrested cells, albeit, with a higher fold induction in HPV^+^ cells. For HPV^-^ SCC19, there was a 1.4-fold induction of the G2/M fraction with radiation alone and a 1.6-fold induction with addition of GA-OH (**Figure S2A**). For HPV^+^ SCC47, the induction of G2/M arrest was even more noticeable. Radiation alone resulted in a 1.7-fold increase in G2/M cells and that number increased to 2.4 when GA-OH was added (**Figure S2A**). Similar findings were observed with additional cell lines, SCC29 and SCC104 (FS2B).These results prompted us to look at the kinetics of the changes of the G2/M fraction by including earlier and later time points. Specifically, we also looked at the changes of cell cycle distribution at 12 and 48 hours in addition to the 24-hr time point. The kinetics study shows that the induction of G2/M arrest at 12 hours is very similar, with HPV^+^ cells demonstrating slightly more induction. However, at 24 hours, a large fraction of HPV^-^ cells is no longer G2/M arrested and at 48 hours, the cells become distributed as are the untreated control cells (**Figure 6C**). In radiation-treated HPV^+^ cells, there is not much difference between the 12 and 24 hr time points as most cells are still G2-M arrested after 24 hrs. At 48 hours, the distribution of the cells starts to slowly shift away from G2/M phase although the distribution remains higher than seen at baseline. The addition of GA-OH makes the effects of radiation much more pronounced at the 24 and 48-hr time points for the SCC47 cells (**Figure 6C**). For SCC19, the impact of GA-OH has on radiation effects is much more attenuated. In summary, HNSCC cell lines treated with IR and GA-OH led were predominantly G2/M arrested with HPV^+^ cells showing a higher fold increase with a greater persistence of cells in the G2/M fraction.

### Effect of radiation and GA-OH on apoptotic induction

**Figure 7:**
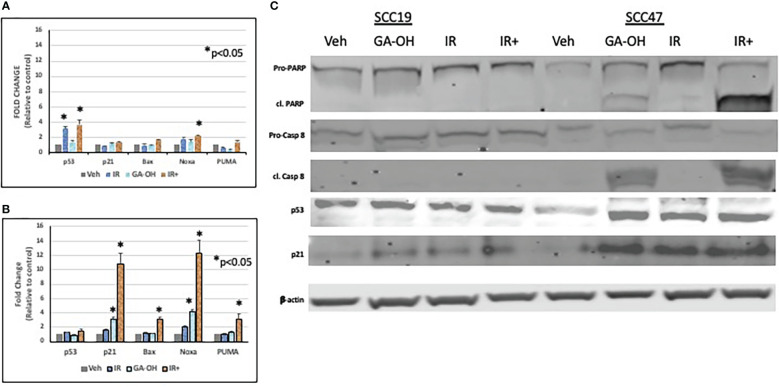
Effect of GA-OH and X-ray radiation on apoptotic markers. SCC19 **(A)** and SCC47 **(B)** cells were treated with GA-OH radiation for 24 hours and expression of p53 target genes was assessed using qPCR. SCC47 has greater expression of downstream targets of p53 compared to SCC19. **(C)** Immunoblotting of various p53 target protein molecules and caspase 8 in both SCC19 and SCC47 cells shows more induction of apoptotic markers in SCC47. cl. PARP stands for cleaved PARP; Pro-Casp 8 stands for pro-Caspase 8, cl. Casp 8 stands for cleaved Caspase 8. *, represents statistical significance of the treatment compared to the vehicle control.

**Figure 8:**
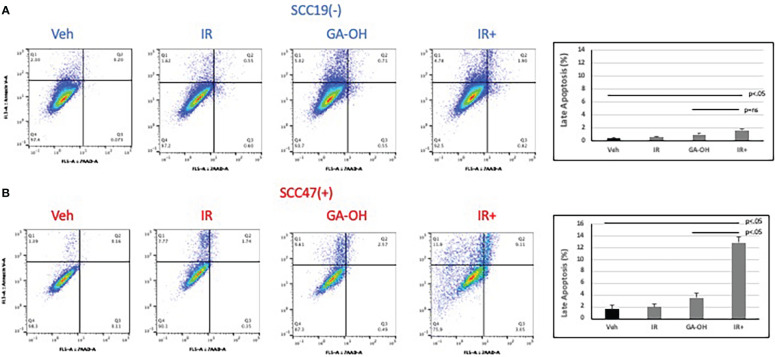
Apoptotic induction after radiation and GA-OH treatment using flow cytometry. SCC19 **(A)** and SCC47 **(B)** cells treated with or without GA-OH were stained with Annexin V and 7AAD 24 hours post-irradiation and analyzed using flow cytometry. SCC47 showed greater apoptotic induction compared to SCC19 particularly in the combination setting. Graphical representation shows quantification of apoptotic cells that are double stained after 24 hrs. Values shown represent means ± standard deviation from 3 independent experiments.

Given the role of GA-OH in apoptosis as described in our previous publication (25), we wondered if GA-OH also enhanced the apoptotic induction of cells following radiation treatment. To evaluate this potential, we utilized qPCR, western blotting and flow cytometry. We first looked at the expression of p53-target genes at the level of mRNA. Previously, we showed that p53 is stabilized at the protein level upon treatment of HPV^+^ cells with GA-OH and that this was also associated with an increase in p21 levels. We now looked at the expression of additional target genes of p53 including three involved in apoptotic induction (Noxa, PUMA, Bax). For SCC19, the highest effect on gene expression of treated cells was seen with p53 itself, particularly with radiation alone and radiation plus GA-OH. Except for Noxa, which had a small but appreciable increase in the combination, the rest of the genes showed incremental changes in expression (**Figure 7A**). For SCC47 (**Figure 7B**), there was a minimal effect on the expression of the p53 gene. However, the effect on all p53 targets was more noticeable, particularly in the cases of p21 and Noxa, and the effects were significantly higher when radiation was combined with GA-OH. Western blotting analysis also showed radiation by itself did not induce significant apoptosis in either cell line (**Figure 7C**). We looked at cleavage of Caspase 8 and PARP as well as induction of p53 and p21 at the protein level. Appreciable cleavage of the apoptotic proteins Caspase 8 and PARP was observed in SCC47 when GA-OH was used alone, with additional cleavage of these proteins noted in the combination setting (**Figure 7C**, FS3). The same can be said for p53 and p21. For SCC19, p53 and p21 induction was observed but no cleavage of Caspase 8 and PARP was seen even in the combination treatment (**Figure 7C**, FS3). Finally, these findings were supported by flow cytometry staining using Annexin IV (a marker of early apoptosis) and 7AAD (a marker of necrosis). Again, radiation by itself did not lead to high apoptotic induction after 24 hours (**Figure 8**). We tested whether radiation by itself induced greater apoptosis at longer time points (48 and 72 hours) but even at these longer time points apoptosis levels were still relatively low (**Supplementary Figure 4**). As with western blotting, the combination of GA-OH with radiation led to more robust increase in apoptosis in SCC47 cells than with either treatment alone. Also notable is not just the observation on cells that are marked for late apoptosis (double staining of Annexin IV and 7AAD) but also the pre-apoptic cells marked with Annexin IV that are higher in combination setting compared to GA-OH alone (**Figures 8A, B**). These data support the contribution of apoptotic induction by GA-OH as a plausible major contributor to the radio-sensitivity of HPV^+^ cells. The HPV^-^ cell line SCC19, the counterpart of SCC47, demonstrated relative radio-resistance and benefitted little from addition of GA-OH. Analysis of apoptosis in additional cell lines SCC29 and SCC104 also showed that apoptosis induction is higher in HPV^+^ cells (FS2C). These observations were also recapitulated in an HPV^-^ primary cell line that we developed from freshly harvested patient tumor cells. Using this cell line, LLU972, we found that again, radiation by itself was not very effective in inducing apoptosis and the combination was not as effective as in HPV^+^ cells. For cell cycle analysis, we noted that the biggest effect of radiation was on the (G2-M) transition, with arrest in this phase happening as previously noted in SCC47 and SCC19 cell lines (**Supplementary Figure 5**). When we performed a dose response analysis of these cells to GA-OH using the MTT assay, we noted that LLU972 displayed a similar sensitivity to GA-OH as did the commercial HPV^-^ cell lines such as SCC19 and was not as sensitive as the HPV^+^ cell lines (**Supplementary Figure 5**). Results with these primary cells are consistent with our main findings that GA-OH is most effective in HPV^+^ cells, with limited activity in HPV^-^ HNSCC cells.

## Discussion

In this study, we have made several key observations with regard to the intrinsic radio response of HPV^+^ cells and the effect of GA-OH on that radiation. Specifically, we have shown that GA-OH influences the cellular response to radiation, and that it enhances the sensitivity of the cells to irradiation even at concentrations that are minimally toxic to the cell. Notably, the radio-sensitization was observed in an HPV-dependent manner as cells with negative HPV status were only weakly affected. DER values ranging from 1.59 to 1.7 were determined for the 3 HPV^+^ cell lines used in this study. We the also studied radiological response of cells to proton beams and again found that HPV^+^ cells were more responsive to addition of GA-OH than were the HPV^-^ cells. HPV^+^ cell lines generally were more radiosensitive to either form of radiation (**Supplementary Figure 1**), with or without GA-OH. This was not surprising and has been well established. Even though a constant RBE of 1.1 is currently used to prescribe proton beam therapy, we found RBE values that varied from this constant and were in all cases higher, which points to the general heterogeneity of tumor cells. Additionally, we found that GA-OH does not enhance the effectiveness of proton beams relative to photons but it enhances the radio-sensitivity of cells to protons compared to protons alone nonetheless.

We further investigated the response of HPV^+^ and HPV^-^ HNSCC cells to DNA damage and their subsequent radiosensitivity. Radiation induces DNA damage and DSBs irrespective of HPV status, but the way the cells deal with the breaks is different. 53BP1 foci appeared to linger somewhat longer in the HPV^+^ cell line, as seen at 24 hours post-irradiation compared to SCC19 (**Figure 4**), although the result did not reach statistical significance. However, by 24 hours we also observed higher levels of apoptosis when GA-OH was added to radiation, and we note that induction of apoptosis of massively damaged cells by the drug may artificially lower the number of observed foci. Nonetheless, the slight enhancement of persistence of 53BP1 foci could imply longer delays in the repair of DSBs and this may in turn contribute to radiosensitivity. Cell cycle analysis results also showed evidence of persistence of unrepaired DSBs through G2/M arrest following irradiation (**Figure 5**), particularly in HPV^+^ cells as previously reported (33, 34). The addition of GA-OH increased the proportion of G2/M-arrested cells even though GA-OH by itself increased both G1 and G2/M cells. The enhancement of radiation-induced G2/M arrest has been previously demonstrated in HPV^+^ cells for agents such olaparib and nirapanib, both PAPR inhibitors (34–36). However, other agents such as cisplatin and Wee1 inhibitors have shown a reduction in the G2/M cells instead (37, 38) implying that more than one cell cycle mechanism can contribute to enhanced radiosensitivity. What is clear, however, is that GA-OH enhances the ability of radiation to induce cell death. We demonstrated this through various approaches, beginning with examination of expression of p53 and caspase 8 and their downstream targets. In our preceding study where we characterized GA-OH from a screen (25), we had found that GA-OH increases the levels of both p53 and caspase 8 activation. In this study, we also found that radiation significantly increased the levels of p53 expression in SCC19 (**Figure 7**). However, when we looked at the downstream targets of p53, there was no concomitant robust expression of those gene targets. This is likely because the p53 in SCC19, like in many HPV^-^ cell lines, is mutant with a frameshift mutation, and therefore unlikely to possess the full transcriptional activation properties of wild type p53 (39). Similarly, we observed weak expression of p53 target genes with immunoblotting in SCC19. In SCC47, we noted remarkable induction of p53 target genes, both at the transcriptional (qPCR) and protein (immunoblotting) levels. Bax, Noxa and PUMA are pre-apoptotic genes, whilst p21 is an effector of cell cycle arrest. Interestingly, GA-OH did not significantly induce p53 itself at the gene level even though immunoblotting demonstrated a significant increase in p53 protein levels. This could be explained by the fact that the wild type gene expression is intact in HPV^+^ cells, while p53 protein levels are attenuated through the degradative actions of E6. In our previous study we found that GA-OH prevents the binding of E6 to E6AP (25), effectively stabilizing the p53 protein from E6-driven proteasome-mediated degradation, consistent with our current finding. We also found that GA-OH activates caspase 8, an effector of the extrinsic apoptotic pathway.

In addition, we observed that radiation by itself did not significantly increase the levels of Caspase 8 cleavage. Combining radiation with GA-OH, however, enhanced the cleavage of caspase 8.

Downstream of caspase 8, the cleavage of both caspase 3 and PARP were increased when radiation was combined with GA-OH, indicating more apoptotic induction. The flow cytometry Annexin V-7AAD assay yielded similar findings, in that radiation did not affect apoptosis significantly. However, adding GA-OH to the radiation treatment caused increased apoptosis by several fold compared to radiation alone. In addition, late apoptosis occurred much quicker in the combination treatment compared to either treatment alone. These results indicate that even though radiation treatment damages the DNA and induces cell cycle arrest at a dose of 4 Gy, it did not lead to significant cell death, at least in the short term. A number of studies have reported the same, showing that apoptosis is not robust in HPV^+^ cells *in vitro* when treated by radiation alone (12, 33, 40, 41). Taken together, our findings indicate that GA-OH may contribute to the radiosensitivity of HPV^+^ cells by delaying resolution of DSBs and enhancing apoptotic induction.

Ultimately, research endeavors aimed at enhancing radiosensitivity of HPV^+^ cells are focused on finding safer treatments for the HPV^+^ HNSCC patient cohort. Continued investigation of the mechanisms of radiosensitivity will be key translationally to develop more effective therapies. Thus far, the radiosensitivity of HPV^+^ tumor cells versus their HPV^-^ counterparts has been attributed to various mechanisms including compromised DNA repair, p53 status, cell cycle regulation, hypoxia and immunogenicity of HPV viral proteins (11, 12, 37, 42–44). Finding agents that selectively harness these mechanisms is an active area of ongoing research (34, 45, 46). Our study adds to these emerging studies, demonstrating that some of the aforementioned factors, particularly DNA repair and p53 expression, can be used to explain the observed effects of GA-OH. We also found that our compound enhanced the radio-response of both protons and photons, a novel result. It is worth noting that the rationale for potentiating radio-sensitivity and thus for improving selectivity and safety is stronger for photon compared to proton radiation. The identification of agents that increase the efficacy of protons will always be welcome, however, particularly in this era of de-escalation. As de-escalated regimens become more and more the focus of HPV-related HNSCC treatment, it is conceivable that targeted therapies will be utilized as less toxic alternatives to cisplatin at some point. At this point it is difficult to definitively say how our findings presented here exactly will move the needle towards the goal of targeted therapy-mediated de-escalation because this study as designed obviously still has limitations and is mostly proof of concept. Further research is warranted to increase the efficacy of E6-targeted inhibitors and to continue to delineate their specific mechanisms in enhancing radio-sensitivity (43). Cisplatin remains the standard radiosensitizer for CRT, due to its relative effectiveness. For this reason, targeted therapies will likely be required to demonstrate effectiveness near to or better than that of cisplatin to be considered as viable alternatives for de-escalation. We showed that cisplatin is better than GA-OH at iso-effective doses although GA-OH is more selective, meaning that higher doses can be afforded before toxicity becomes an issue. Looking at it from this angle, the selectivity of GA-OH and other targeted therapies may make it possible to reach the effectiveness currently seen with cisplatin. Cetuximab has thus far been regarded as the most promising cisplatin substitute for HNSCC treatment in conjunction with radiation. However, in various recent clinical trials, cetuximab has shown inferior survival outcomes as compared to cisplatin, and this has dampened the hope of incorporating its use into de-intensified regimens for HPV-related HNSCC (15–18). Together, these observations further underscore the need for greater efforts to discover and develop more robust and selective targeted agents. In our case, we plan to work towards this goal of robustness by following up these observations we have made in this study in the future. We are aware that this study has a number of limitations and that what we have reported is only proof of principle. We are particularly interested in pursuing animal studies for *in vivo* evaluation of GA-OH and its interaction with radiation. Specifically, we plan on testing the combination of GA-OH and radiation in an SCC47 xenograft and/or patient-derived xenograft models to establish efficacy as well as other important vital parameters such as pharmacokinetics and toxicity of GA-OH. We also intend on further exploring some of the differences noted between proton and photon radiation in HPV^-^ and HPV^+^ cell lines both *in vitro* and *in vivo*.

## Data availability statement

The original research contributions presented in the study are included in the article/**Supplementary Material**. Further inquiries can be directed to the corresponding author.

## Author contributions

LC and PD-H contributed to conception of the study. LC, PD-H, MV, AB, JU contributed to the experimental design of the study. LC, MV, AB performed the experiments. SL provided access to HNSCC cancer tissues. LC wrote the first draft of the manuscript. All authors contributed to the article and approved the submitted version.
