# Supplementary material for: GA-OH enhances the cytotoxicity of photon and proton radiation in HPV+ HNSCC cells
Source: Front Oncol. 2023 Feb 10;13:1070485. doi: 10.3389/fonc.2023.1070485 (PMC9950506; doi:10.3389/fonc.2023.1070485)
Supplement: Supplementary file 1 [file Presentation_1.pptx]

## Slide 1
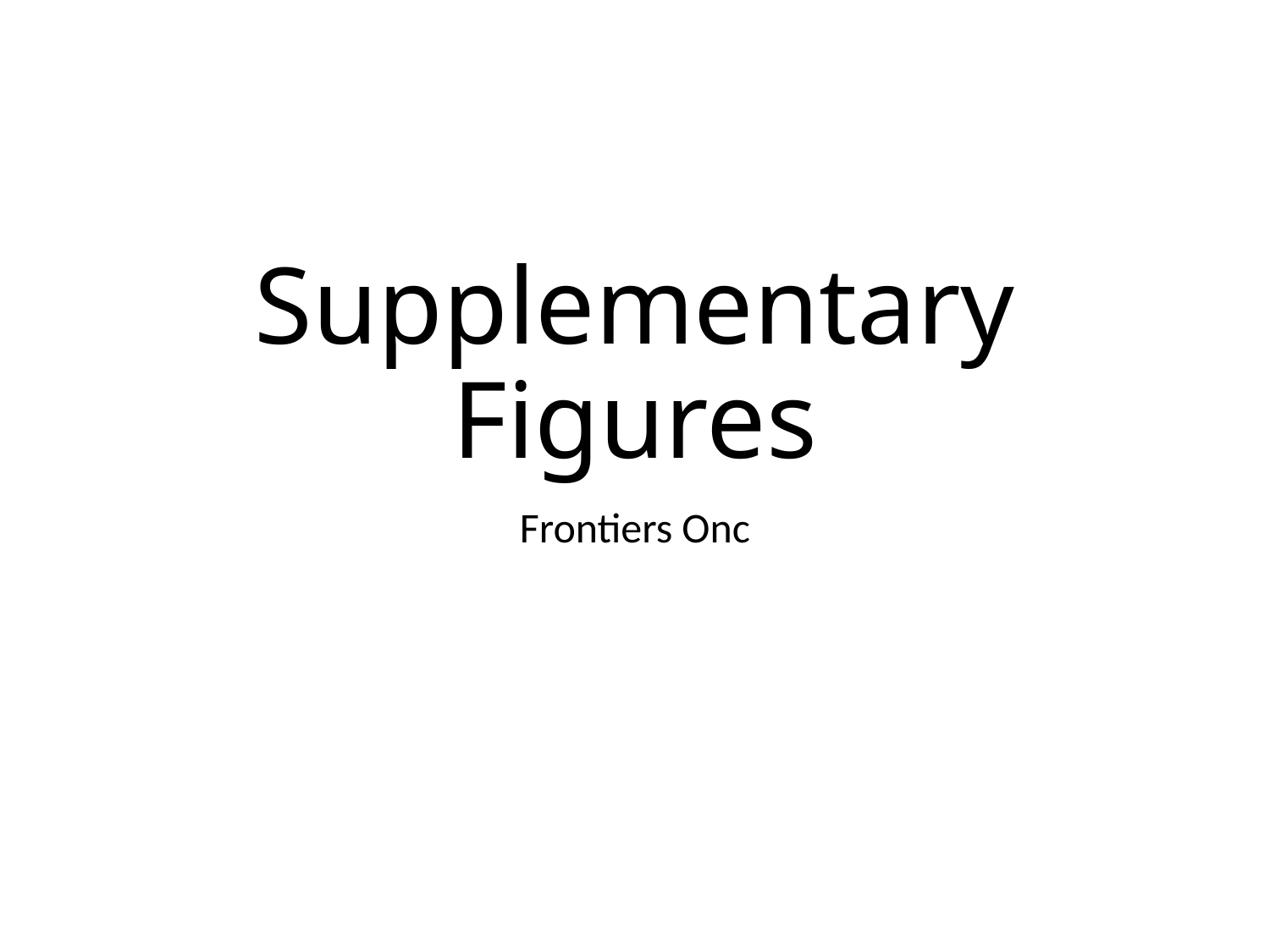

# Supplementary Figures
Frontiers Onc

## Slide 2
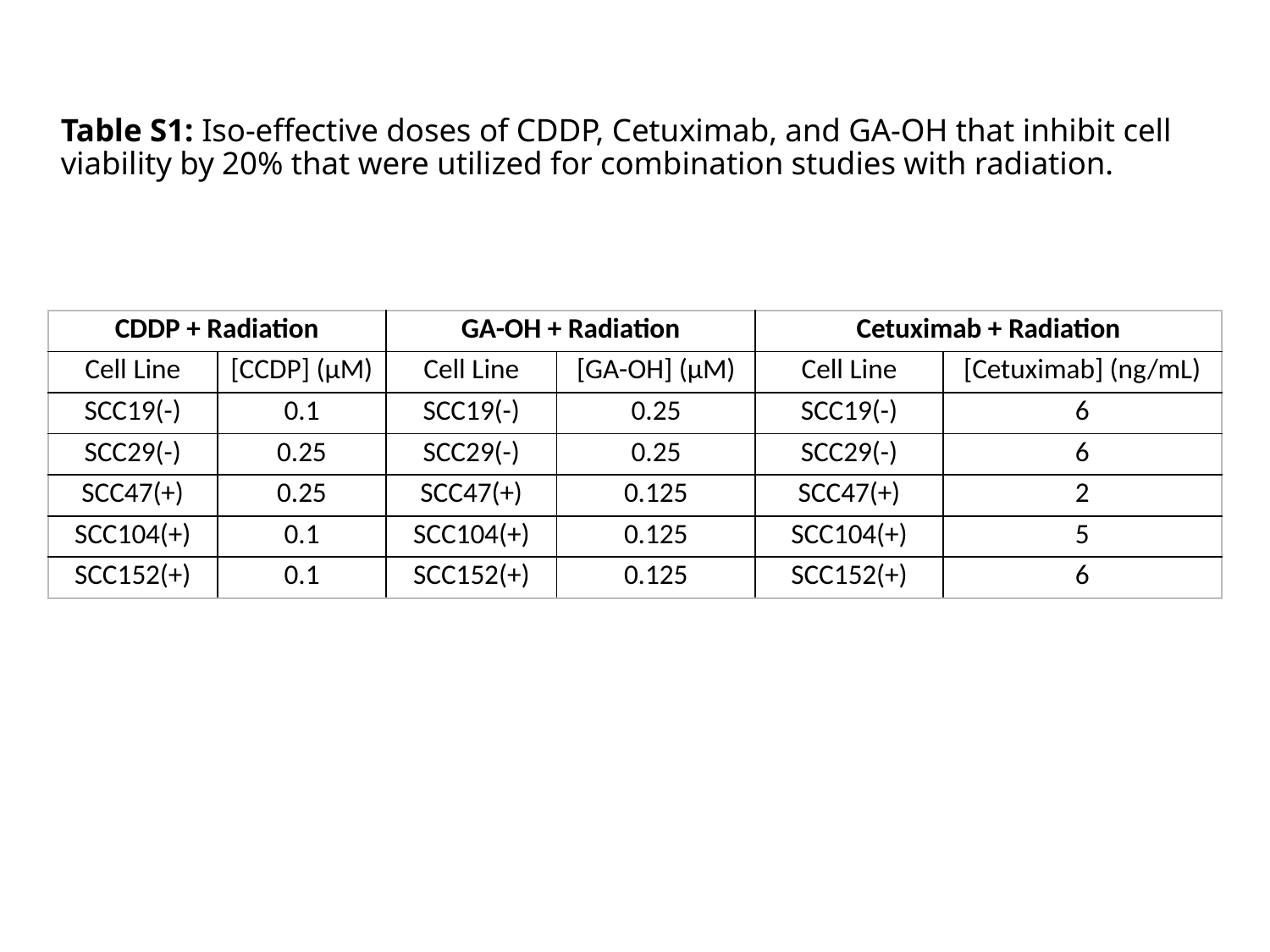

# Table S1: Iso-effective doses of CDDP, Cetuximab, and GA-OH that inhibit cell viability by 20% that were utilized for combination studies with radiation.
| CDDP + Radiation | | GA-OH + Radiation | | Cetuximab + Radiation | |
| --- | --- | --- | --- | --- | --- |
| Cell Line | [CCDP] (µM) | Cell Line | [GA-OH] (µM) | Cell Line | [Cetuximab] (ng/mL) |
| SCC19(-) | 0.1 | SCC19(-) | 0.25 | SCC19(-) | 6 |
| SCC29(-) | 0.25 | SCC29(-) | 0.25 | SCC29(-) | 6 |
| SCC47(+) | 0.25 | SCC47(+) | 0.125 | SCC47(+) | 2 |
| SCC104(+) | 0.1 | SCC104(+) | 0.125 | SCC104(+) | 5 |
| SCC152(+) | 0.1 | SCC152(+) | 0.125 | SCC152(+) | 6 |

## Slide 3
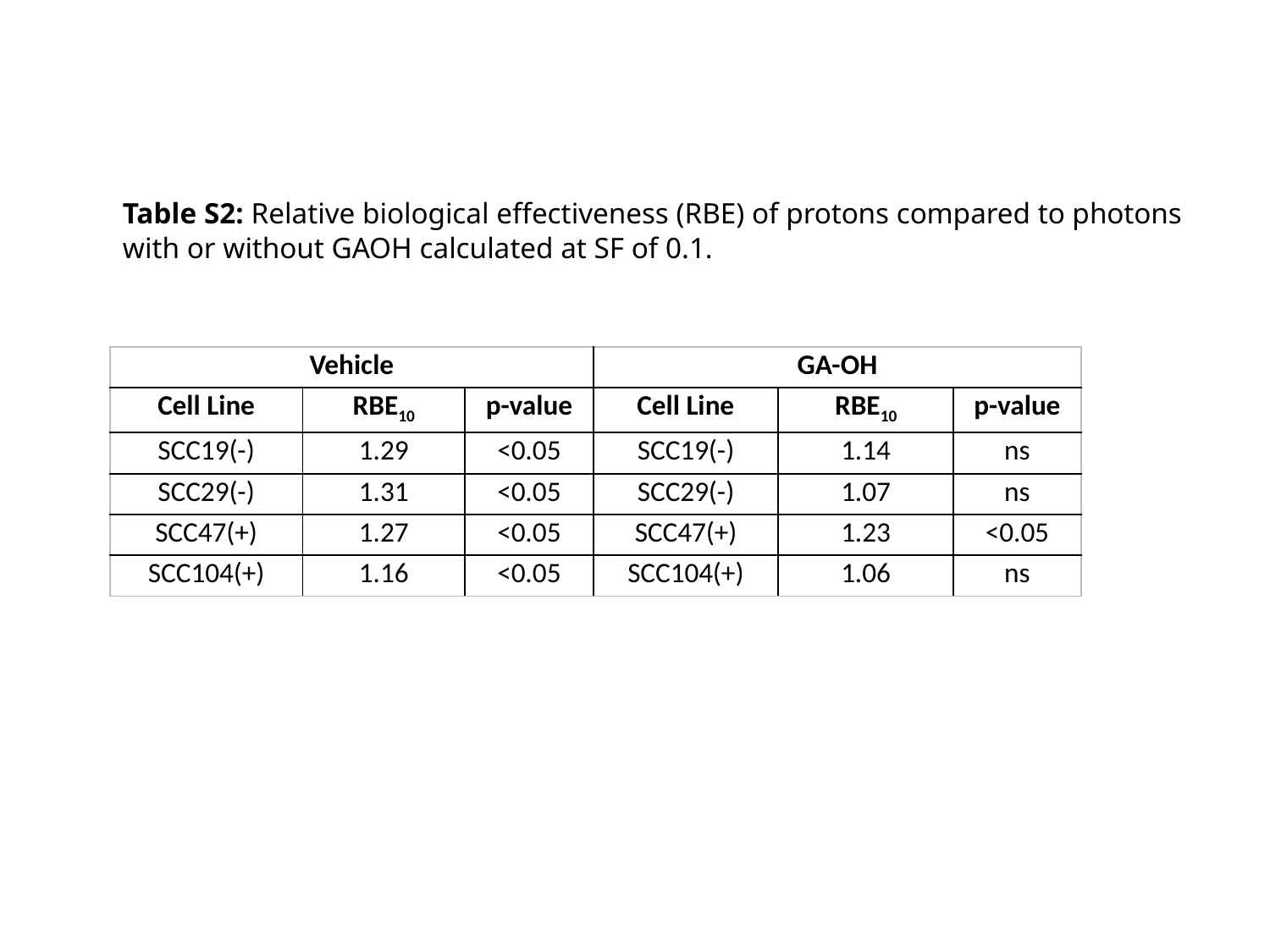

# Table S2: Relative biological effectiveness (RBE) of protons compared to photons with or without GAOH calculated at SF of 0.1.
| Vehicle | | | GA-OH | | |
| --- | --- | --- | --- | --- | --- |
| Cell Line | RBE10 | p-value | Cell Line | RBE10 | p-value |
| SCC19(-) | 1.29 | <0.05 | SCC19(-) | 1.14 | ns |
| SCC29(-) | 1.31 | <0.05 | SCC29(-) | 1.07 | ns |
| SCC47(+) | 1.27 | <0.05 | SCC47(+) | 1.23 | <0.05 |
| SCC104(+) | 1.16 | <0.05 | SCC104(+) | 1.06 | ns |

## Slide 4
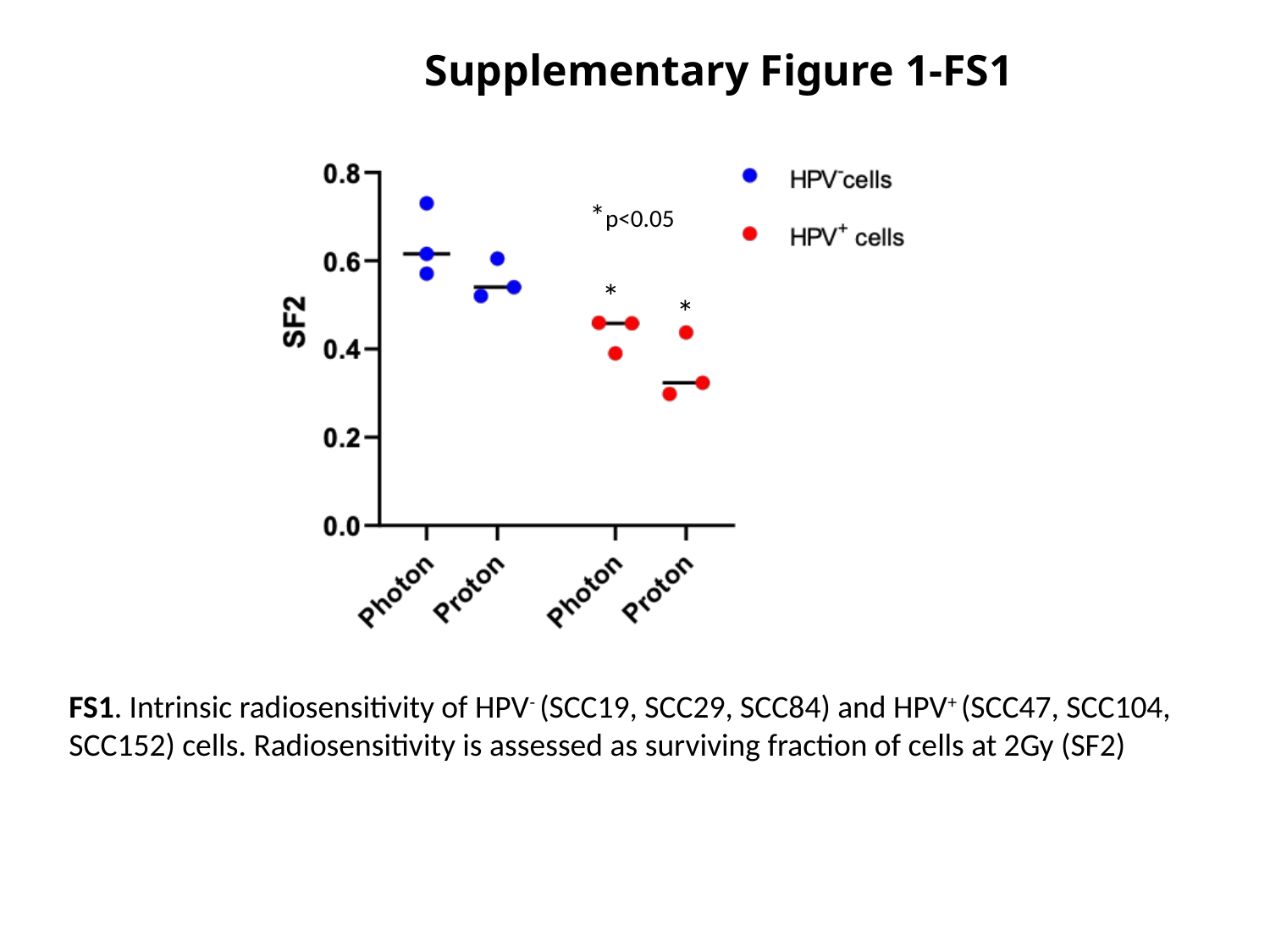

# Supplementary Figure 1-FS1
 *p<0.05
*
*
FS1. Intrinsic radiosensitivity of HPV- (SCC19, SCC29, SCC84) and HPV+ (SCC47, SCC104, SCC152) cells. Radiosensitivity is assessed as surviving fraction of cells at 2Gy (SF2)

## Slide 5
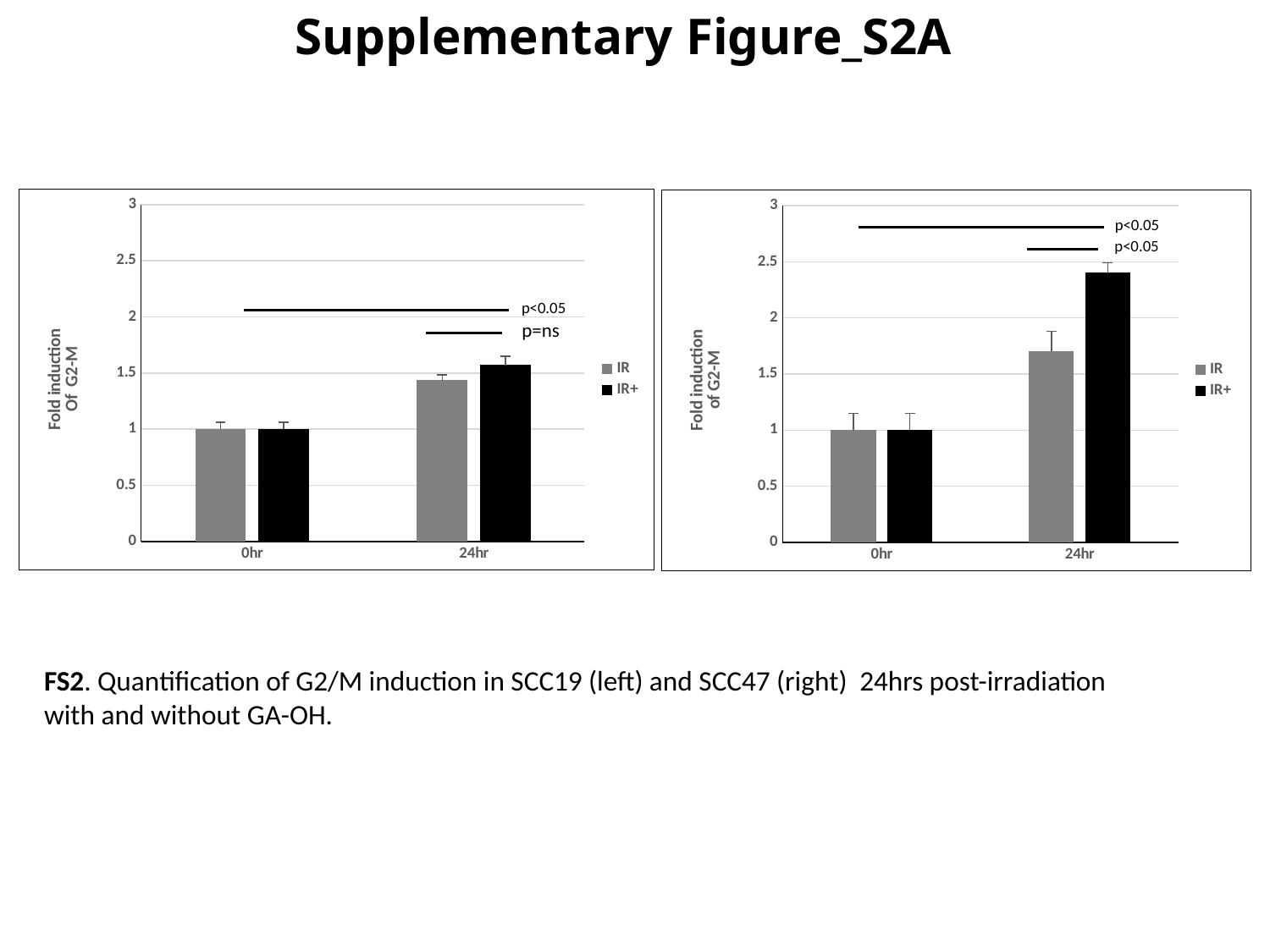

# Supplementary Figure_S2A
### Chart
| Category | IR | IR+ |
|---|---|---|
| 0hr | 1.0 | 1.0 |
| 24hr | 1.432708688245315 | 1.5689948892674614 |p<0.05
p=ns
### Chart
| Category | IR | IR+ |
|---|---|---|
| 0hr | 1.0 | 1.0 |
| 24hr | 1.7 | 2.4 |p<0.05
p<0.05
FS2. Quantification of G2/M induction in SCC19 (left) and SCC47 (right) 24hrs post-irradiation with and without GA-OH.

## Slide 6
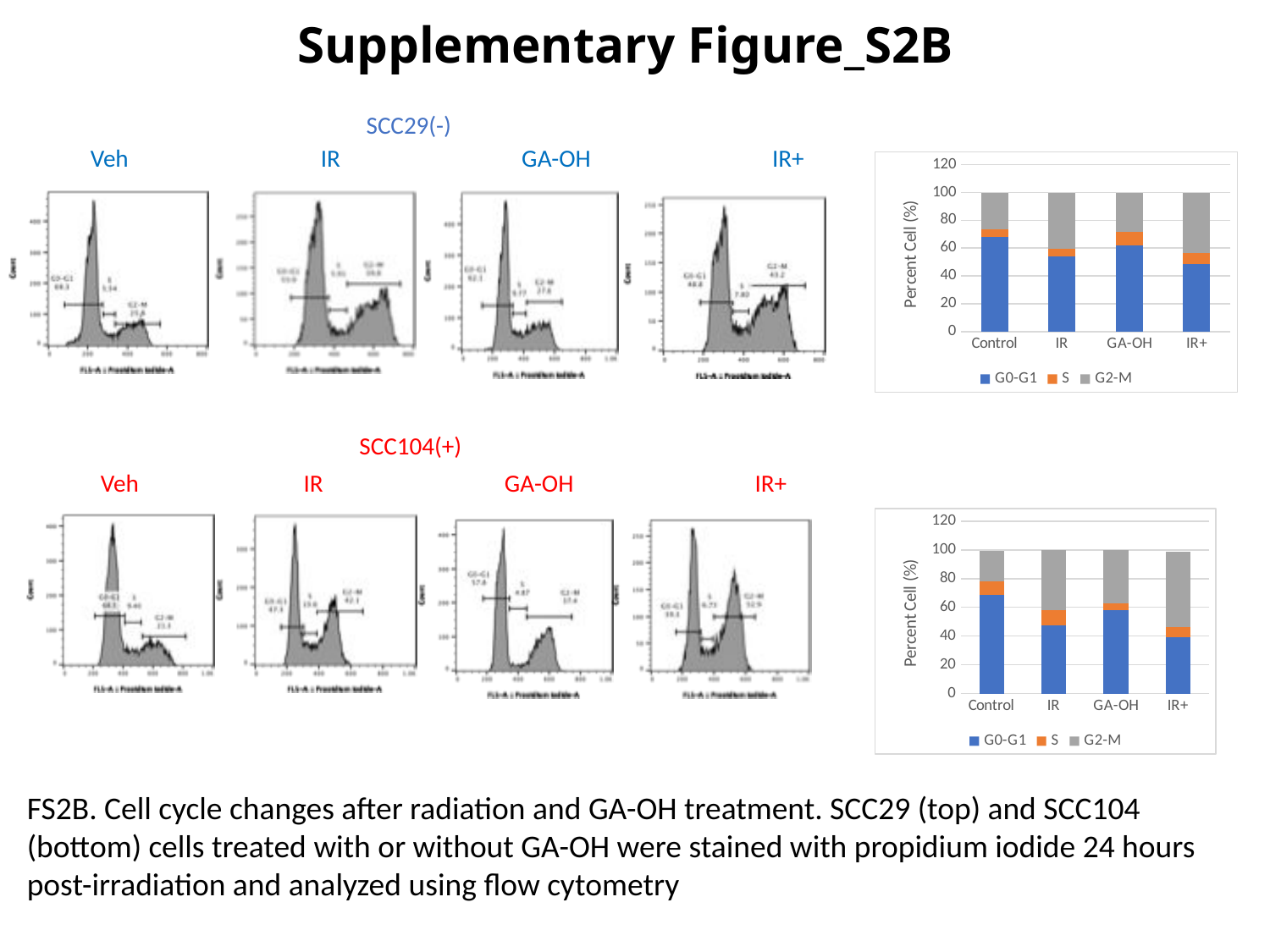

# Supplementary Figure_S2B
SCC29(-)
 Veh IR GA-OH IR+
### Chart
| Category | G0-G1 | S | G2-M |
|---|---|---|---|
| Control | 68.1 | 5.54 | 25.8 |
| IR | 53.9 | 5.91 | 39.8 |
| GA-OH | 62.1 | 9.76 | 27.6 |
| IR+ | 48.8 | 7.8 | 43.2 |
SCC104(+)
 Veh IR GA-OH IR+
### Chart
| Category | G0-G1 | S | G2-M |
|---|---|---|---|
| Control | 68.5 | 9.4 | 21.3 |
| IR | 47.3 | 10.6 | 42.1 |
| GA-OH | 57.8 | 4.87 | 37.4 |
| IR+ | 39.3 | 6.73 | 52.9 |
FS2B. Cell cycle changes after radiation and GA-OH treatment. SCC29 (top) and SCC104 (bottom) cells treated with or without GA-OH were stained with propidium iodide 24 hours post-irradiation and analyzed using flow cytometry

## Slide 7
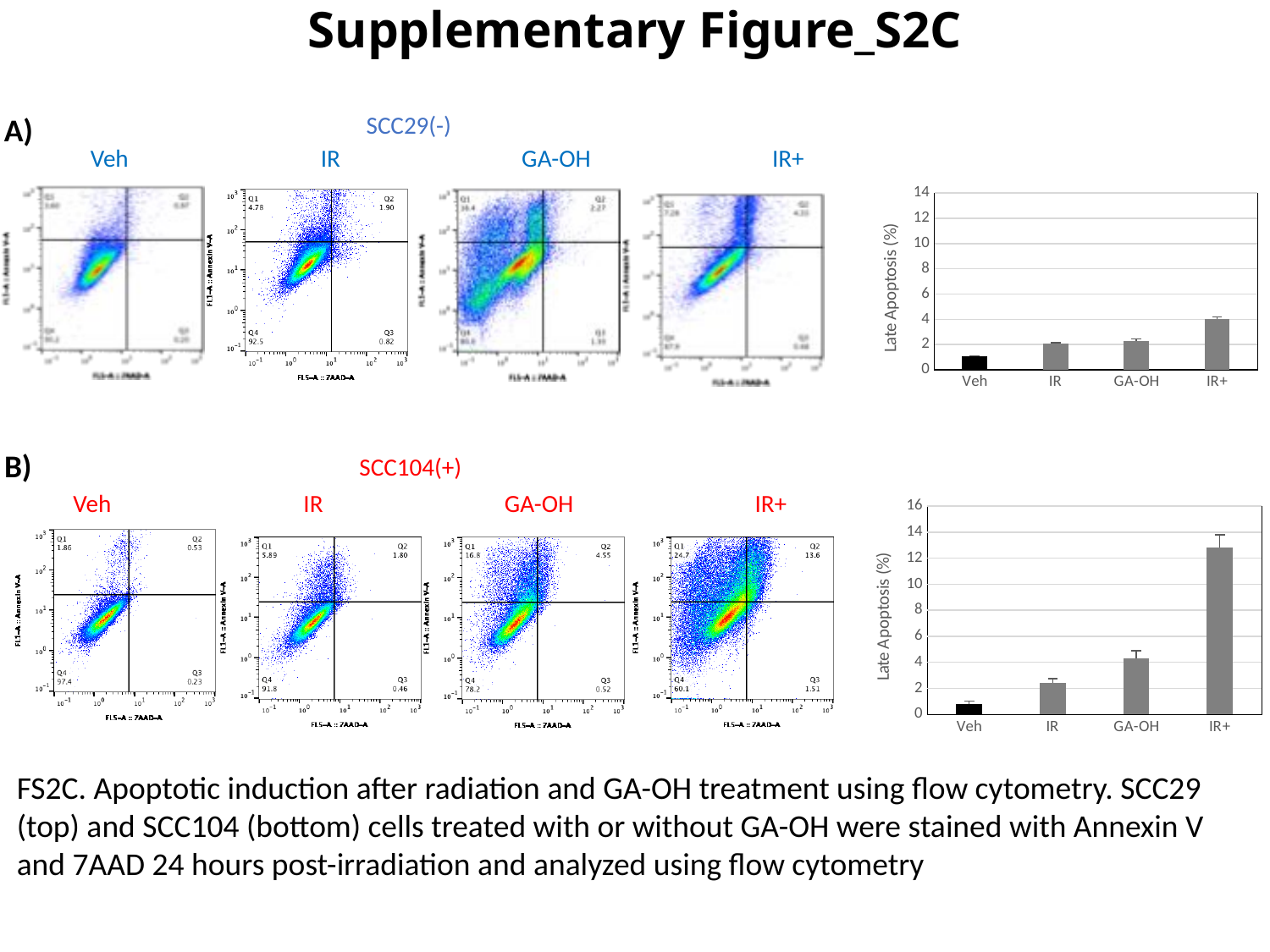

# Supplementary Figure_S2C
SCC29(-)
A)
 Veh IR GA-OH IR+
### Chart
| Category | |
|---|---|
| Veh | 1.1 |
| IR | 2.06 |
| GA-OH | 2.3 |
| IR+ | 4.0 |
B)
SCC104(+)
 Veh IR GA-OH IR+
### Chart
| Category | |
|---|---|
| Veh | 0.824 |
| IR | 2.45 |
| GA-OH | 4.28 |
| IR+ | 12.8 |
FS2C. Apoptotic induction after radiation and GA-OH treatment using flow cytometry. SCC29 (top) and SCC104 (bottom) cells treated with or without GA-OH were stained with Annexin V and 7AAD 24 hours post-irradiation and analyzed using flow cytometry

## Slide 8
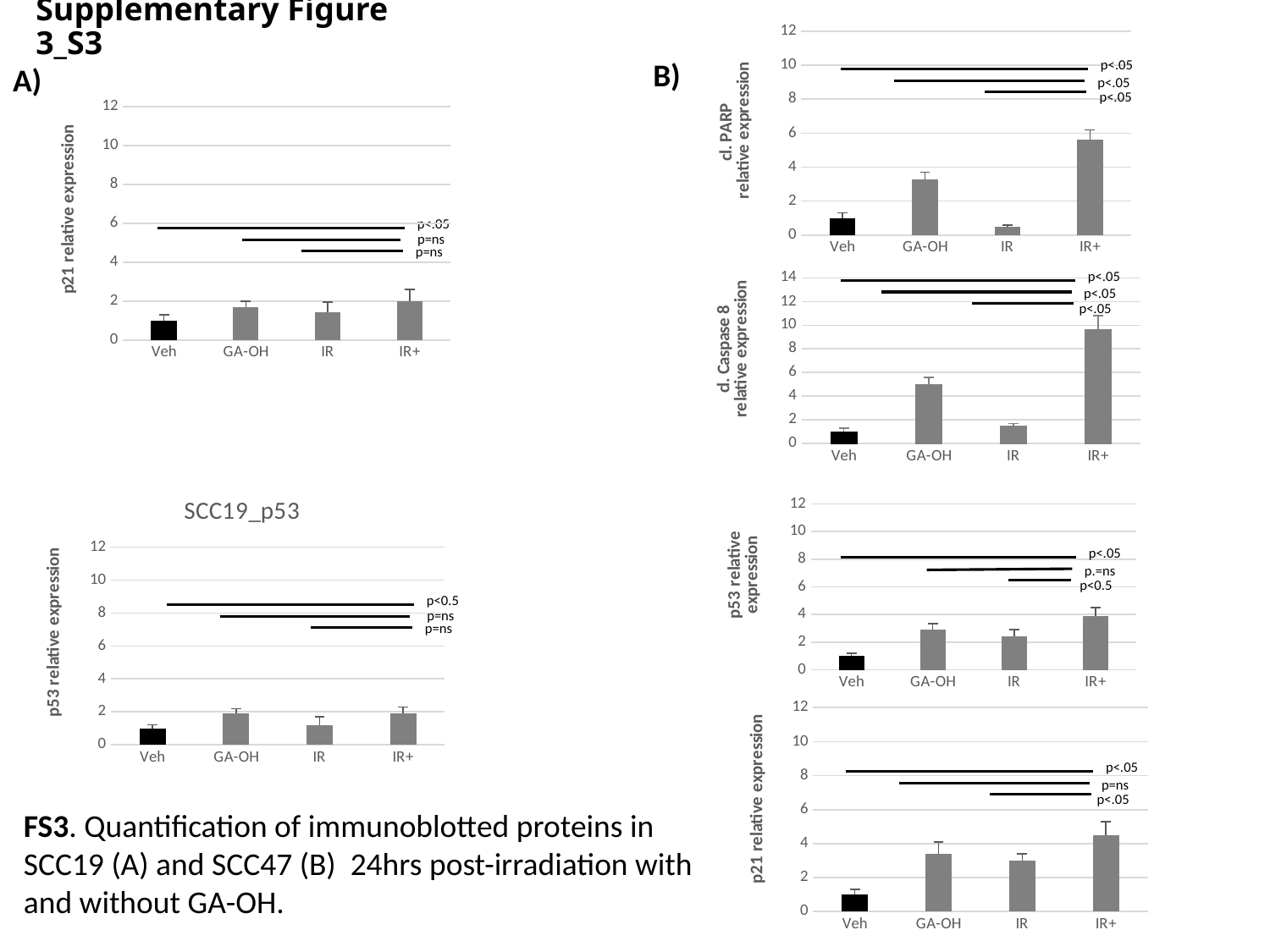

# Supplementary Figure 3_S3
### Chart
| Category | |
|---|---|
| Veh | 1.0 |
| GA-OH | 3.3 |
| IR | 0.5 |
| IR+ | 5.6 |B)
p<.05
A)
p<.05
p<.05
### Chart
| Category | |
|---|---|
| Veh | 1.0 |
| GA-OH | 1.7 |
| IR | 1.45 |
| IR+ | 2.01 |p<.05
p=ns
p=ns
p<.05
### Chart
| Category | |
|---|---|
| Veh | 1.0 |
| GA-OH | 5.0 |
| IR | 1.5 |
| IR+ | 9.6 |p<.05
p<.05
### Chart: SCC19_p53
| Category | |
|---|---|
| Veh | 1.0 |
| GA-OH | 1.9 |
| IR | 1.2 |
| IR+ | 1.88 |
### Chart
| Category | |
|---|---|
| Veh | 1.0 |
| GA-OH | 2.93 |
| IR | 2.4 |
| IR+ | 3.9 |p<.05
p.=ns
p<0.5
p<0.5
p=ns
p=ns
### Chart
| Category | |
|---|---|
| Veh | 1.0 |
| GA-OH | 3.4 |
| IR | 3.0 |
| IR+ | 4.5 |p<.05
p=ns
p<.05
FS3. Quantification of immunoblotted proteins in SCC19 (A) and SCC47 (B) 24hrs post-irradiation with and without GA-OH.

## Slide 9
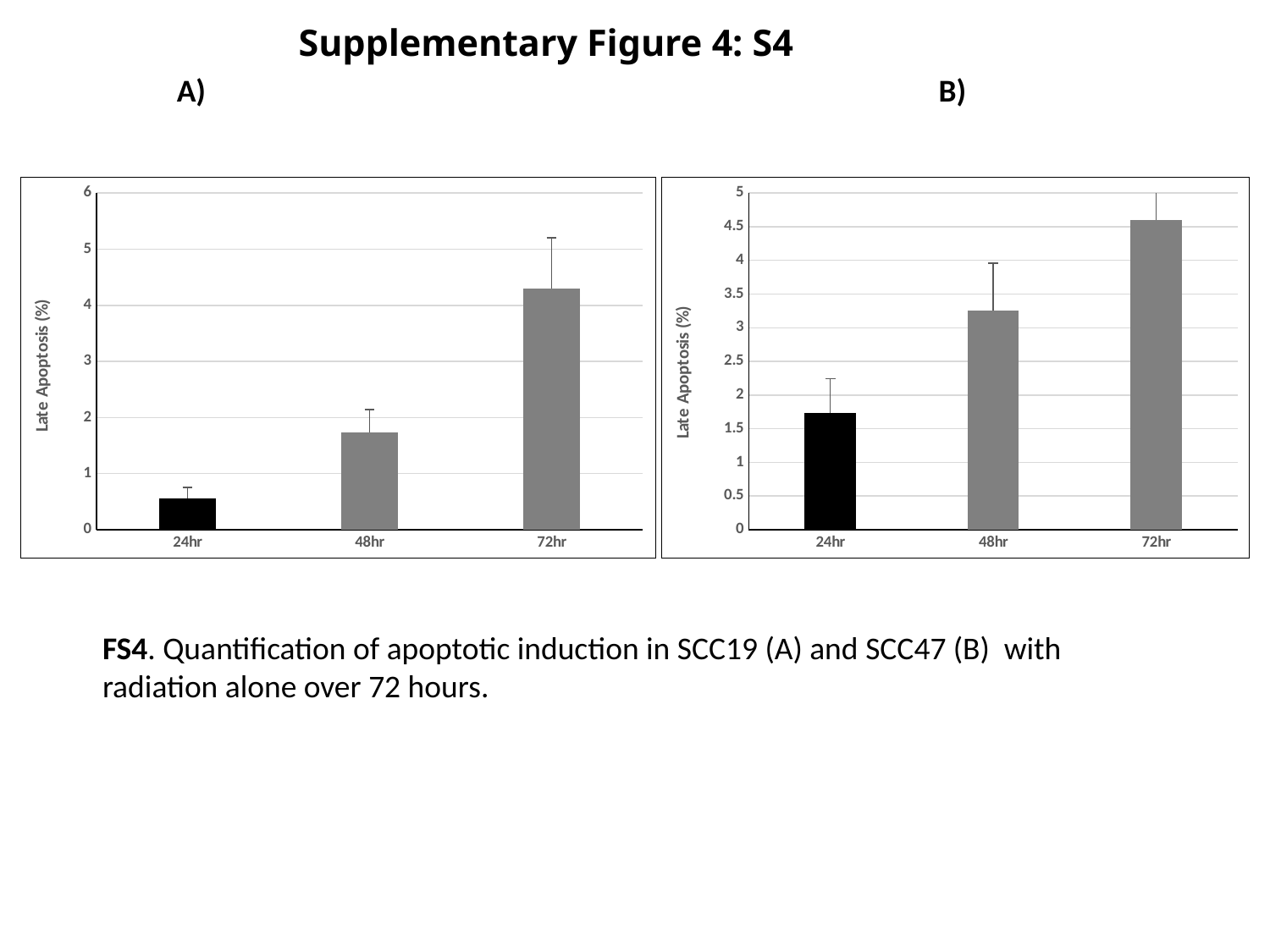

# Supplementary Figure 4: S4
A)
B)
### Chart
| Category | |
|---|---|
| 24hr | 0.55 |
| 48hr | 1.74 |
| 72hr | 4.3 |
### Chart
| Category | |
|---|---|
| 24hr | 1.74 |
| 48hr | 3.26 |
| 72hr | 4.6 |FS4. Quantification of apoptotic induction in SCC19 (A) and SCC47 (B) with radiation alone over 72 hours.

## Slide 10
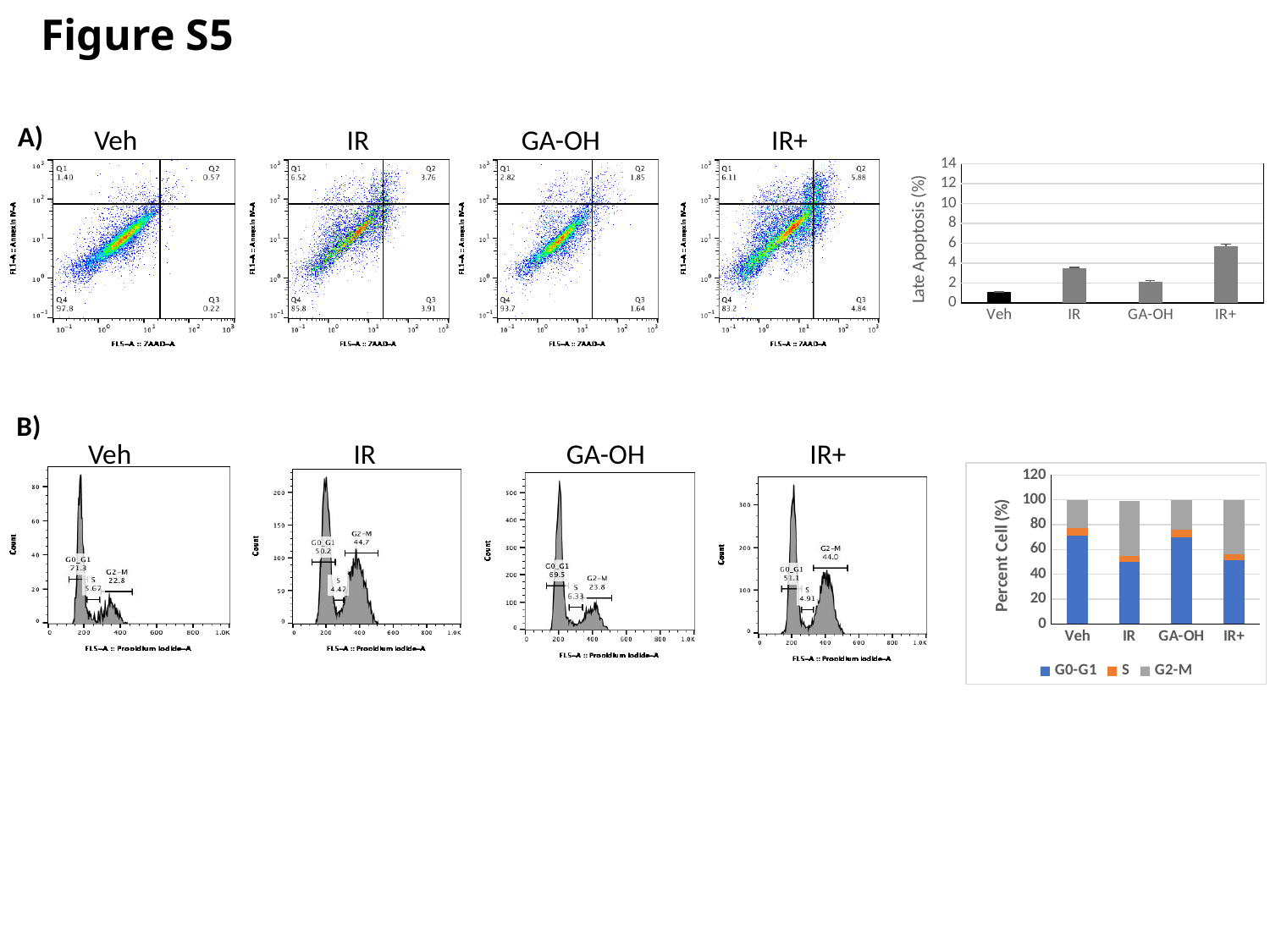

# Figure S5
A)
 Veh IR GA-OH IR+
### Chart
| Category | |
|---|---|
| Veh | 1.1 |
| IR | 3.5 |
| GA-OH | 2.1 |
| IR+ | 5.7 |B)
Veh IR GA-OH IR+
### Chart
| Category | G0-G1 | S | G2-M |
|---|---|---|---|
| Veh | 71.3 | 5.67 | 22.8 |
| IR | 50.2 | 4.47 | 44.7 |
| GA-OH | 69.5 | 6.33 | 23.8 |
| IR+ | 51.1 | 4.91 | 44.0 |

## Slide 11
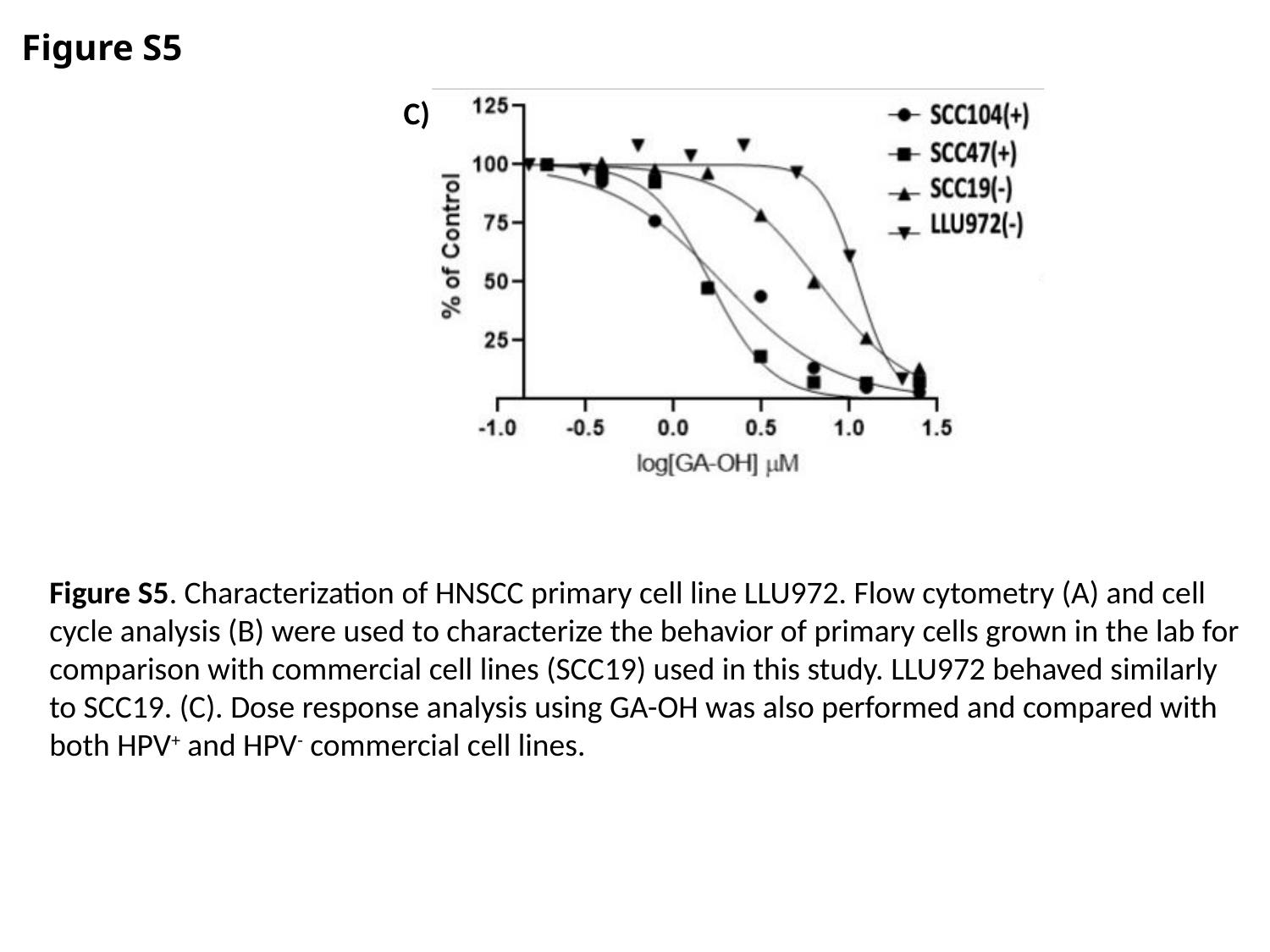

# Figure S5
C)
Figure S5. Characterization of HNSCC primary cell line LLU972. Flow cytometry (A) and cell cycle analysis (B) were used to characterize the behavior of primary cells grown in the lab for comparison with commercial cell lines (SCC19) used in this study. LLU972 behaved similarly to SCC19. (C). Dose response analysis using GA-OH was also performed and compared with both HPV+ and HPV- commercial cell lines.
